# Adrenal venous sampling in a patient with adrenal Cushing syndrome

**Published:** 2015-06-30

**Authors:** Carlos Esteban Builes-Montaño, Carlos Andrés Villa-Franco, Alejandro Román-Gonzalez, Alejandro Velez-Hoyos, Santiago Echeverri-Isaza

**Affiliations:** 1 Médico Internista Endocrinólogo. Sección de Endocrinología, Departamento de Medicina Interna. Hospital Pablo Tobón Uribe - Universidad de Antioquia. Medellin, Colombia; 2 Médico Internista. Departamento de Medicina Interna, Hospital Pablo Tobón Uribe. Medellin, Colombia; 3 Residente de Endocrinología. Sección de Endocrinología, Departamento de Medicina Interna. Universidad de Antioquia. Medellin, Colombia; 4 Patólogo. Departamento de Patología. Hospital Pablo Tobón Uribe. Medellin, Colombia; 5 Médico Radiólogo Intervencionista. Departamento de Radiología. Hospital Pablo Tobón Uribe. Medellin, Colombia

**Keywords:** Cushing syndrome, adrenal cortex diseases, adrenal cortex function Tests

## Abstract

The primary bilateral macronodular adrenal hyperplasia or the independent adrenocorticotropic hormone bilateral nodular adrenal hyperplasia is a rare cause hypercortisolism, its diagnosis is challenging and there is no clear way to decide the best therapeutic approach. Adrenal venous sampling is commonly used to distinguish the source of hormonal production in patients with primary hyperaldosteronism. It could be a useful tool in this context because it might provide information to guide the treatment. We report the case of a patient with ACTH independent Cushing syndrome in whom the use of adrenal venous sampling with some modifications radically modified the treatment and allowed the diagnosis of a macronodular adrenal hyperplasia.

## Abstract (es)

La hiperplasia macro nodular bilateral o hiperplasia adrenal nodular bilateral independiente de la hormona adrenocorticotrópica es una de las causas menos frecuentes de hipercortisolismo, su diagnóstico supone un reto y no se tiene claridad de cuál es la mejor aproximación terapéutica. El muestreo venoso adrenal que frecuentemente se utiliza para hacer la distinción del sitio de producción hormonal en el hiperaldosteronismo primario podría ser una herramienta útil en este contexto ya que podría brindar información que pudiera guiar el tratamiento. Presentamos el caso de una paciente con síndrome de Cushing ACTH independiente en quien el uso del muestreo venoso adrenal con algunas modificaciones cambio de manera radical el tratamiento y permitió confirmar una hiperplasia adrenal macro nodular.

## Introduction

Cushing syndrome is a rare disease caused in most cases (after steroid use has been excluded) by a pituitary ACTH-producing adenoma 1. However, sometimes it is caused by an adrenal condition such as a unilateral adrenal adenoma, adrenal carcinoma or bilateral adrenal hyperplasia either pigmented nodular adrenocortical disease, also known as bilateral micronodular hyperplasia, or bilateral adrenal macronodular hyperplasia 2-4. The standard of care in the adrenal Cushing's syndrome is the resection of the affected gland, unilateral adrenalectomy in the case of an adenoma or bilateral in the case of a hyperplasia. In the latter case it implies a lifetime glucocorticoid and mineralocorticoid supplement. Some patients can present with a bilateral adrenal adenoma, in the case of primary hyperaldosteronism most of the time only one of the lesions is responsible of the hormonal production and the other one is simply a non-producing adenoma. In these patients with clearly identified hyperaldosteronism a bilateral adrenal veins catheterization with hormonal sampling is sometimes necessary in order to locate the source of aldosterone production, which may be unilateral or bilateral regardless of history and imaging findings 5,6. This procedure has been described very rarely to differentiate cases of adrenal Cushing syndrome. In this report we describe the first case of adrenal vein catheterization for the study of an adrenal Cushing's syndrome and a review of the bilateral adrenal hyperplasia as a rare cause of this disease.

## Case report

**Figure 1.**
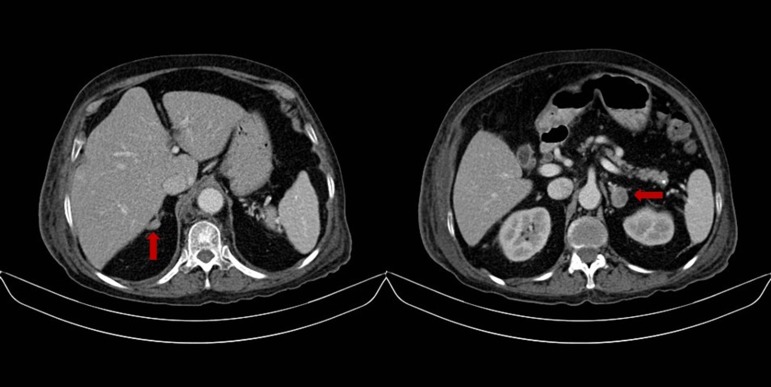
Abdominal tomography. Right adrenal nodule 14x9 mm and left adrenal gland nodule 23x18 mm.

A 76 years-old woman with history of controlled hypertension (with losartan and amlodipine) presented to our hospital referring weight loss of 4 kg in two months, edema that progressed to anasarca and back lumbar pain. Besides the edema her physical examination was completely normal with no clinical signs suggestive of hypercortisolism. Among the studies requested, an abdominal tomography showed a right adrenal gland nodule of 14x9 mm and another one in the left adrenal gland of 23x18 mm (Fig. 1), additionally multiple vertebral fractures were reported. As part of the study of adrenal adenomas the patient had an abnormal value of cortisol after a low dose suppression test with dexamethasone (13.3 µg/dL (normal value: <1.8 µg/dL), with a normal value of free urinary cortisol and her potassium was low (she was not on diuretics). The results of the patient laboratory test are shown on Table 1.

**Table 1.**
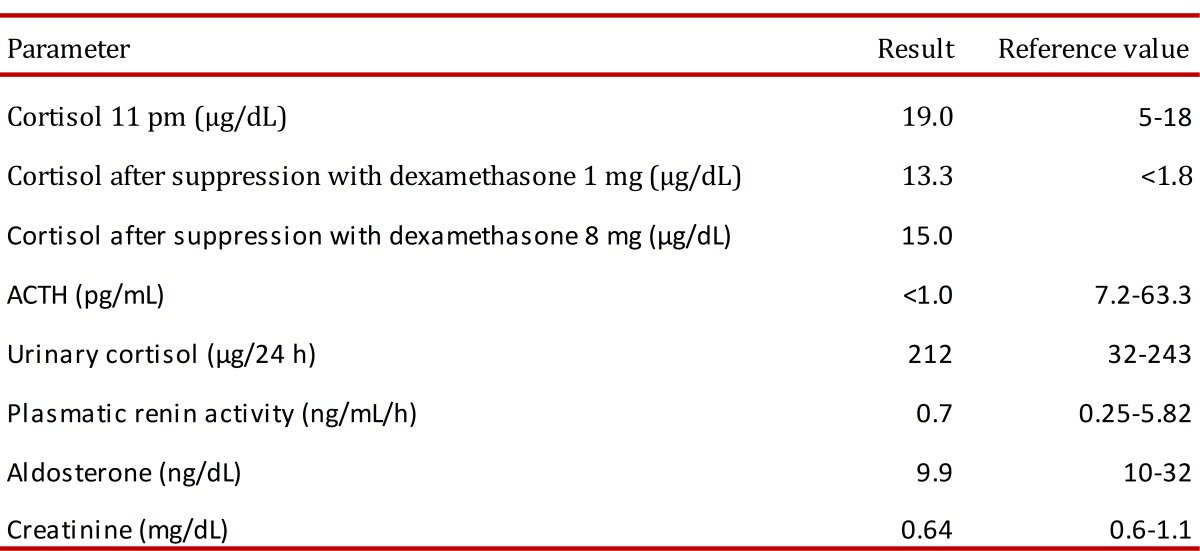
Main laboratory results.

With these results an ACTH (adrenocorticotropic hormone) independent form of hypercortisolism was diagnosed and because the scan of the abdomen had documented the presence of two adenomas, one in each adrenal gland larger than 10 mm, we found ourselves with a therapeutic challenge since the resection of one of the glands may not cure the hypercortisolism in case that the hormonal production came from both glands and the resection of both glands with leave the patient with a permanent hypoadrenalism. It was decided then to perform an adrenal venous sampling to try to determine the origin of the of cortisol production.

**Figure 2.**
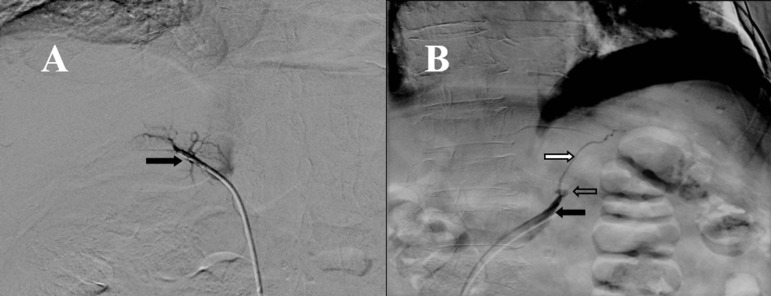
Adrenal vein sampling. **A**: Right adrenal venography using 4Fr multipurpose catheter from femoral approach, where adrenal central vein can be seen right (black arrow) with glandular branches that converge to it. **B**: Left adrenal venography using multipurpose catheter 4Fr from femoral approach, where common adrenal trunk (black arrow) can be seen with the catheter in the interior, where the lower left phrenic vein (white arrow) and left adrenal vein (arrow without filler) converge.

A previously described protocol was used 7 with some changes. The authors of the original protocol propose the measurement of epinephrine as a method to determine the proper location of the catheters when performing the sampling. Because we do not have readily available the measurement of plasma catecholamines the test was performed using radiographic documentation of the tip of the catheter and aldosterone levels were used to make the corrections in the dilution between both sides 2. Samples of both adrenal veins and inferior cava vein were taken (the procedure is shown in Figure 2 and the results are shown in Table 2). Based on the model of interpretation of adrenal venous sampling proposed by Young 3 the test suggest that the patient has an adrenal hyperplasia with predominance in the production of cortisol from the right side, the results were discussed with the patient and the surgical team and it was then decided to perform a bilateral adrenalectomy. The result of the histological study of the glands was consistent with a bilateral adrenal macronodular hyperplasia.

**Table 2.**
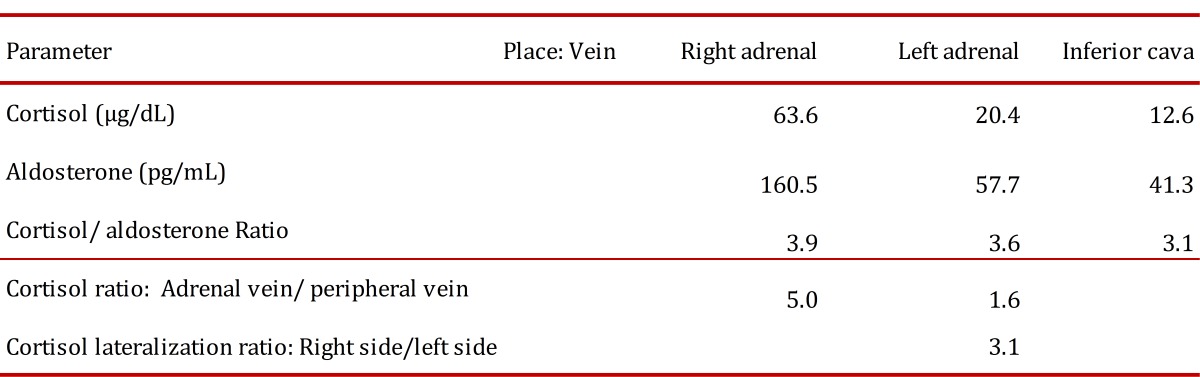
Adrenal venous sampling results.

The patient did not have any postoperative complication and received replacement therapy with hydrocortisone 50 mg every 8 h during the first 48 h and subsequently received prednisolone 10 mg and 0.1 mg of fludrocortisone replacement with resolution of the hypokalemia.

## Discussion

The macronodular bilateral adrenal hyperplasia or ACTH-independent macronodular adrenal hyperplasia (AIMAH), is one of the rarest causes of hypercortisolism and its true frequency has not been established. Less than 2% of all cases of hypercortisolism are explained by any form of bilateral adrenal hyperplasia either macro or micro nodular 4. AIMAH usually presents with bilateral lesions that are larger than 10 mm and it is more frequent in women. The onset is usually later when compared to other forms of hypercortisolism 8 (usually after the fifth decade of life) and the clinical manifestations are generally an autonomous subclinical glucocorticoids hypersecretion or subclinical Cushing syndrome (SCS). Our patient met the two criteria proposed for the diagnosis of this entity 9 The SCS is a diagnostic challenge due to the lack of usual clinical features of this condition, the occasional small elevations of cortisol levels, the great amount of tests, the different cutoff points proposed by different authors for different tests and the lack of specificity of radiological characteristics that can occur in different conditions besides AIMAH like adrenal metastases. Our patient had lost of the circadian rhythm of cortisol production evidenced by high levels of cortisol at 23:00 h and autonomy in the production shown by the ACTH levels and lack of suppression in the low-dose dexamethasone test. As has been reported by other authors urinary free cortisol has a poor diagnostic yield in patients with SCS. Our patient had completely normal levels in several measurements 10.

Adrenal vein sampling is used mainly in patients with hyperaldosteronism. Even in high volume centers with experienced radiologist the success rates are around 70 to 90%6. Adrenal vein sampling is rarely used for the study of hypercortisolism and this is probably due to the rarity of cases in which adrenal cortisol secretion and bilateral nodules present together, but it can be a useful test in this group of patients. It is proposed that some patients could be treated with a unilateral adrenalectomy as production of cortisol could be related to the size of the nodule 11. But in the case of our patient catheterization revealed something else entirely, the nodule of the left adrenal gland was much larger but the production of cortisol came predominantly from the right adrenal gland nodule, although it has been reported that the 85% of people may have an increased production of cortisol in the right adrenal gland patient values exceeded the gradient described 5.

Based on the above it was decided to perform the extraction of both adrenal glands and the result of the pathology confirmed the diagnosis of AIMAH.

## Conclusion

To our knowledge this is the first report of Adrenal vein sampling in a patient with hypercortisolism and bilateral adrenal adenomas in our country.

We made some changes to the procedure reported in the literature, given the difficulty with the measurement of catecholamines we chose to perform verification of proper positioning of the catheter tip by the image from the venography and to correct the dilution difference by measuring aldosterone, the biochemical diagnosis was confirmed histological which allows us to conclude that the verification by venography was adequate.

This technique allowed an accurate diagnosis and avoids the need for re-intervention in the case that the glandular size guided the treatment.
